# Etiology of anxious and fearful behavior in juvenile neuronal ceroid lipofuscinosis (CLN3 disease)

**DOI:** 10.3389/fpsyt.2023.1059082

**Published:** 2023-04-11

**Authors:** John R. Ostergaard

**Affiliations:** Department of Child and Adolescence, Centre for Rare Diseases, Aarhus University Hospital, Aarhus, Denmark

**Keywords:** juvenile neuronal ceroid lipofuscinosis, CLN3, neurodegenerative diseases, anxiety, fear, Paroxysmal Sympathetic Hyperactivity

## Abstract

**Background:**

Juvenile neuronal ceroid lipofuscinosis (JNCL, CLN3) is a childhood-onset neurodegenerative disease with prominent symptoms comprising a pediatric dementia syndrome. As in adult dementia, behavioral symptoms like mood disturbances and anxiety are common. In contrast to in adult dementia, however, the anxious behavioral symptoms increase during the terminal phase of JNCL disease. In the present study, the current understanding of the neurobiological mechanisms of anxiety and anxious behavior in general is addressed as will a discussion of the mechanism of the anxious behavior seen in young JNCL patients. Based on developmental behavioral points of view, known neurobiological mechanisms, and the clinical presentation of the anxious behavior, a theory of its etiology is described.

**Result and discussion:**

During the terminal phase, the cognitive developmental age of JNCL patients is below 2 years. At this stage of mental development individuals act primarily from a concrete world of consciousness and do not have the cognitive ability to encounter a normal anxiety response. Instead, they experience the evolutionary basic emotion of fear, and as the episodes typically are provoked when the adolescent JNCL patient is exposed to either loud sounds, is lifted from the ground, or separated from the mother/known caregiver, the fear can best be perceived as the developmental natural fear-response that appears in children 0-2 years of age. The efferent pathways of the neural fear circuits are mediated through autonomic, neuroendocrine, and skeletal-motor responses. The autonomic activation occurs early, is mediated through the sympathetic and parasympathetic neural systems, and as JNCL patients beyond puberty have an autonomic imbalance with a significant sympathetic hyperactivity, the activation of the autonomic nervous system results in a disproportionate high sympathetic activity resulting in tachycardia, tachypnea, excessive sweating, hyperthermia, and an increased atypical muscle activity. The episodes are thus phenotypically similar to what is seen as Paroxysmal Sympathetic Hyperactivity (PSH) following an acute traumatic brain injury. As in PSH, treatment is difficult and so far, no consensus of a treatment algorithm exists. Use of sedative and analgesic medication and minimizing or avoiding provocative stimuli may partly reduce the frequency and intensity of the attacks. Transcutaneous vagal nerve stimulation might be an option worth to investigate rebalancing the sympathetic-parasympathetic disproportion.

## Introduction

Juvenile neuronal ceroid lipofuscinosis (JNCL, CLN3) is a childhood-onset neurodegenerative disease with prominent symptoms comprising a pediatric dementia syndrome with intellectual decline, loss of adaptive skills, and mood and behavioral impairments. In the Northern European countries the incidence is 2-5 per 100,000 (1, 2). Clinically, the onset of symptoms occurs at 5–7 years of age. Although the temporal sequence of symptom onset varies, the most common symptom families recognize early on is vision loss, rapidly progressing to blindness. Symptom progression continues with loss of cognition, motor skills, speech, development of epilepsy, and emergence of behavioral and mood problems. The disease course unfolds slowly, with an average survival of 15–20 years from symptom onset to the end of life (3). The occurrence of behavioral impairments follow an inverse U-shaped curve in which the symptoms initially worsen as disease advances, before dropping off in the later stages of the disease (4). So far, there is no established treatment that can stop, reverse, or prevent the disease (3).

The first formal characterization of general neurobehavioral features of JNCL was conducted in Finland in the 1990s by Santavuori and coworkers (5). High rates of physical restlessness (35%), aggression (40%), fear (45%), and sleep problems (30%) were reported and 10% also experienced psychotic symptoms (5). Already in the latter half of the 1970s, however, Sorensen & Parnas (6) described the presence of a special behavioral symptom where adolescent JNCL patients show a frightened facial expression, accompanied by excessive sweating, increased body temperature, tachycardia, tachypnea, and increased motor activity of arms, legs or other parts of the body (6). Identical behavioral phenomena were described in 1993 in a Dutch residential setting for patients with JNCL (7). Due to a similar semiology, it has been suggested that these attacks of anxiety/anxious behavior and increased atypical motor activity occurring in adolescent JNCL patients might represent a clinical event similar to Paroxysmal Sympathetic Hyperactivity (PSH) most often seen following acute traumatic brain injuries (8).

Behavioral symptoms, including anxiety, are also very common in adult dementia syndromes like Alzheimer and Parkinson's disease (9, 10). In Alzheimer, anxiety is relatively stable across the range of dementia severity, until the profound and terminal stages, where the occurrence of anxiety decreases (9), similar to the described inverse U-shaped occurrence of the general behavioral symptoms observed in JNCL (4). This is unlike the anxiety/anxious behavior seen in adolescent JNCL patients, where the anxious behavior increases in frequency and severity with increasing age (6–8, 11) and even may be related to early death (11). Therefore, the neuro-etiological mechanism is most probably different from the causal mechanisms of the commonly occurring anxiety that we see in otherwise healthy people and in adult dementia syndromes. In the present study, the current understanding of the neurobiological mechanisms of anxiety and anxious/fearful behavior in general will be addressed, as will a discussion of the possible etiology and mechanism of the anxious behavior seen in young JNCL patients.

## Anxiety and fear

Anxiety represents a state of heightened arousal, enhanced vigilance, and general feelings of averseness in the absence of an immediate threat. Fear is an emotional response to a known or definite threat and is most probably the strongest of our primitive instincts for survival (12). In general, the emotional states are adaptive and promote avoidance to protect individuals from potential dangers. From an evolutionary perspective, anxiety is considered as a psychological hazard-detection system, and under conditions of uncertainty, excessive anxiety is usually less costly to fitness than insufficient anxiety. Thus, the human anxiety response is to prepare the individual to detect and deal with threats (13). Data from human studies suggest that a person's anxiety level is due to an interaction between genetic and environmental factors occurring early in life. Important is both a generalized and a specific psychological vulnerability focused on particular events or circumstances during early life (14). Fear is one of three distinct emotions that are present from birth; the other two are anger and joy, and children can use a distinct facial expression to express these emotions in an appropriate context already after 2 months of age (15). They can recognize their primary caregiver (often the mother) by sight around 5 months of age, and between 6 and 12 months effective attachment relationships are established and learning of fear is partly mediated by the reactions of the caregivers. When an infant see a stranger it looks at the mother (caregiver) frequently to monitor her response (16). If she smiles, this reassures; if she shows alarm, this augment the infant's fear, and fear of strangers emerges. Fear of separation/being alone also appears during the last half of the 1st year. Height fear starts in infants shortly before they start crawling at 6–8 months of age and rises with crawling experience. As the 2-year old child explores further afield, fear of animals is developed (16). Worth noting, even fear as an emotional category almost exclusively refers to negatively valenced experiences fear nonetheless denotes a variety of psychological states that vary as a function of sensory input, context, and previous experience (17). One of the subtypes of fear is called *recreational fear* which is defined as a mixed emotional experience of fear and enjoyment, where humans derive pleasure from playful engagement with fear-inducing situations. Such engagement ranges from mildly scary children's activities, such as playfully being chased by a parent, to full blown horror media like horror films and haunted attractions (18, 19). By simultaneous examination of subjective and physiological parameters, an inverted-U-shaped curve can be demonstrated, in which amusement and fear can coexist in frightening leisure activities as enjoyable until the activities offer forms of arousal dynamics that are “just right”, and beyond this point, fear takes over (19).

## Neurobiological aspects of fear and anxiety

For some authors, fear and anxiety are indistinguishable whereas others believe that they are distinct phenomena because anxiety is a generalized response to an *unknown* or *internal* conflict, and can only be understood by taking into account some of its cognitive aspects, whereas fear is focused on *known* or “real”, *external* danger. The distinction does not exclude some overlap in underlying brain and behavioral mechanisms, and current evidence from clinical and preclinical research suggests that anxiety and fear arise from alterations in a set of highly interconnected neural circuits, of which the amygdala, ventral hippocampus, and medial prefrontal cortex are key nodes (20–22). The efferent pathways of the anxiety-fear circuit are mediated through autonomic, neuroendocrine, and skeletal-motor responses. The autonomic activation is among the earlier psychophysiological response observed and is produced by the sympathetic and parasympathetic neural systems. Activation of the sympathetic system produces increases in blood pressure, heart rate, sweating, and pupil dilation and through the parasympathetic nervous system the metabolic demands are suppressed. The hypothalamo-pituitary-adrenocortical axis triggers or facilitates the endocrine (primarily catecholamines) and neuropeptide release, whereas the regulatory control of the skeletal muscle response is more complex, depending on whether subtle movements involving a few muscle groups of the facial muscles are essential or whether freezing of the body or escape and fight is required (20, 23).

Studies have shown that low base-line parasympathetic activity in toddlers (24 months of age) predisposes to increased fear, and that parental appraisal of children's fearfulness predicts parenting behavior like overprotection and over solicitousness that even reinforce the children's fearful behavior (24, 25). In addition, recent studies have shown that a feeling of fear is contagious, i.e., can spread from one person to another through a dynamic coupling of physiological synchrony between a “demonstrator's” and the “observer's” autonomic nervous system (26).

## The clinical presentation of anxious behavior in adolescent JNCL

The recurring periods of non-epileptic episodes of severe anxious behavior with concomitant increased muscle activity are the clinical manifestation that gives rise to most concern for parents and caregivers of adolescent patients with JNCL (8). Beyond a frightened facial expression while clinging to the bed or chair, the young JNCL patients demonstrate increased atypical muscle activity, excessive sweating, increased body temperature and blood pressure, tachycardia, tachypnea and often show non-sustained multi-directional nystagmus, pupil dilation and a decreased awareness (6–8). The episodes might start without obvious reason, but typically the episodes occur when the patients are being left alone, exposed to stimuli that are either non-nociceptive (lifting the patient from bed to a wheelchair, loud sounds) or only minimally nociceptive (bathing, brushing teeth) (6–8). Similar recurrent episodes have also been described in a 22 year old patient with JNCL following a case of status epilepticus (27). In this patient, the episodes lasted from 10 s−5 min and occurred as frequently as every 10 s in clusters. When each episode ceased, the muscles relaxed, heart rate and blood pressure normalized, the diaphoresis resolved, and the consciousness improved (27). In a retrospective review of medical records, similar episodic symptoms were identified in 10 of 21 subjects (mean age 21.2; range 10–39 years of age) in a recently published Norwegian case series (11). In these patients, episodes of agitation, restlessness, grimacing, and other unspecific motor symptoms were reported from the mean age of 17 years (range: 14–27). Consistent with previously published descriptions (6–8), the rate of episodes increased during the later stage of the disorder and lasted up to hours and days, and a non-epileptic etiology was documented by electro-encephalographic recordings in many, although not all of the cases (11).

## Etiological considerations

### Can adolescent or adult patients with CLN3 encounter anxiety?

It is generally accepted that emotional and cognitive processes cannot be dissociated, and that cognitive apprehension is critically involved in emotional experiences as well as in coping strategies (20). Thus, when discussing the behavioral patterns of adolescent JNCL patients it is necessary to include their current intellectual abilities. We are dealing with a condition that occurs in a phase of JNCL where the patients are largely without intelligible verbal language, most are significantly physically affected and need help with most daily tasks such as food and liquids intake, cleanliness, visits to the toilet, and if not already the case, many will soon become bedridden or totally dependent of a wheel-chair. Cognitively, they have problems remembering what just happened, their daily routines, planned events, even of great personal significance, and remembering and maintaining their strong interests (28). Thus, during this stage of disease, the JNCL patients both physically and mentally have a functional level corresponding to stage (6)−7 of the primary degenerative dementia scale of Reisberg and coworkers (29).

The particular combination of symptoms in JNCL—dementia and loss of vision and speech intelligibility—is a challenge and places limits on intellectual assessments beyond 17 years of age as many intelligence tests evaluate both verbal and visual reasoning skills, and even some verbal tasks such as picture naming require visual identification (4). Additionally, vision loss also excludes tests of non-verbal/visual reasoning, and loss of speech intelligibility eventually also limits the child's ability to provide verbal responses to language-based tasks. However, it is well documented that the cognitive decline starts early and proceeds very quickly. Already at the time of diagnosis, comprehension, short-term memory and attention are weakened by most, and by the age of 10 years, the total IQ has fallen by about 50 percent (30). Thus, although we do not have validated test results for total IQ and intellectual age coefficients for JNCL patients beyond the age of 17 years, the available literature (4, 28, 30, 31) justifies an estimate that assumes the intellectual age of JNCL patients in the current context of anxious behavior equivalent to a child of ~1½-2 years of age. At this stage of development the affected individual acts primarily from a concrete world of consciousness and will not have the cognitive ability to show a qualified response to an “*unknown*” or “*internal conflict*”, as otherwise characterizes anxiety and anxiety disorders in adults or children beyond an intellectual age of 5-6 years of age. Thus, adolescent and adult patients with JNCL cannot encounter anxiety.

### Can the episodes be perceived as natural fear responses?

The outburst of the anxious behavior is typically provoked when adolescent or older JNCL patients are exposed to separation from parents or known caregivers, meet unknown people, hear loud sounds, or are lifted from bed to a chair, i.e., during episodes which from an developmental point of view are equivalent to episodes that trigger a developmental “natural fear”—response in a mentally 1–2 year old child (16, 32). As the episodes cannot represent a real anxiety it is reasonable instead to perceive them fear responses, and as the episodes are related to identical stimuli and concrete threats as the developmental fear response that emerges during the first 1–2 years of life (32), it seems justified to perceive the episodes of the anxious behavior as natural occurring fear responses.

If the assumption that the non-epileptic periodic attacks of severe anxious behavior are to be understood as a developmental natural fear phenomenon, we need to explain the dramatic clinical picture it presents when it occurs in adolescent or older JNCL patients who are faced with general discomfort or an allodynic stimulation (33). By using heart-rate variability (HRV) as a proxy for the activity of the autonomic nervous system, a significant age-dependent decrease in the parasympathetic activity has been documented in patients with JNCL, whereas no age related change of the neural and neuro-hormonal sympathetic activity occurs (8, 34). The decrease of the parasympathetic activity takes place throughout all age groups and the activity is rather low beyond 18–20 years of age (8). Thus, when an adolescent JNCL patient, having a developmental cognitive level equal to a 1–2 years old child, experiences the sensation of fear, the markedly reduction in parasympathetic activity without any accompanying change in sympathetic activity leads to an autonomic imbalance with a significantly increased sympathetic activity. As activation of the autonomic neural system is one of the earliest psychophysiological responses triggered within the efferent fear pathway, this imbalance results in an increase in blood pressure, heart rate, respiratory frequency, excessive sweating, hyperthermia, and pupil dilation. Additionally, the endocrine and motor systems are activated, and the fear reaction is then phenotypically quite similar to the episodes which normally occurs following an acute traumatic or anoxic brain injury (35–38), known as Paroxysmal Sympathetic Hyperactivity (PSH) (39).

## Therapeutic considerations

Treatment of PSH related to traumatic brain injury includes medications and attempt to minimize or avoid provocative situations. To date, no randomized clinical trials have been performed and no consensus exists for a treatment algorithm. Unfortunately, none of the medications are supported by solid evidence; however, clinical experience supports their value and the cumulative, albeit limited, evidence (37, 39) suggests that first line oral medications include benzodiazepines (midazolam, clonazepam) and potent analgesia (37, 39). Gabapentin is often used to treat neuropathic pain, and in a single case series study in PSH following severe traumatic brain injury, gabapentin reduced or prevented the paroxysms allowing an overall reduction in other medications without recurrence of symptoms (40). Sympathetic outflow may be reduced by α-2 adrenergic agonists, non-selective β-blockers, and certain neuromodulators including bromocriptine may reduce temperature and sweating, whereas baclofen can reduce spasticity and spasms (39). Of note, dopamine antagonists (such as haloperidol or chlorpromazine) are not useful to treat PSH and may even worsen the disorder and cause other serious side effects (41, 42).

In adolescent and young JNCL patients, the paroxysms most often occur as allodynic responses to external stimuli or internal triggers like intestinal discomfort. It is therefore very important to avoid or at least to minimize these fear-provoking situations. That includes appropriate positioning, use of weighted blankets, prevention of constipation/bladder retention, infections, unprepared and rapid movements, loud noises, and to ensure a close but calming physical presence of parents and/or health care workers. As feeling of fear is contagious (27), i.e., can spread from one person to another, it is an important preventive approach that the staff do not show or express their own fear or concern of the patient's clinical condition having the patient nearby. When the episodes occur for no apparent reason intestinal discomfort or infections should be suspected. No clinical trials have been performed and no consensus exists for a treatment algorithm in PSH related to JNCL, but as in PSH induced by acute brain trauma, the first line oral medications include benzodiazepines (midazolam, clonazepam) and analgesia. Gabapentin may increase the threshold for intestinal discomfort or allodynic pain, and sympathetic outflow may be reduced by α-2 adrenergic agonists and non-selective β-blockers.

Recent studies have shown that coping strategies of fear learning and fear extinction in healthy children requires a well-functioning vagal alertness (43). Additionally, differences in parasympathetic tone accompany changes in pain perception, both in health and disease, in such a way that reduced parasympathetic activity increases visceral pain (44). So far, we do not have enough knowledge of the bi-directional functional state of the vagal nerve in adolescent JNCL patients, but transcutaneous vagal nerve stimulation might be an option worth to investigate rebalancing the sympathetic-parasympathetic disproportion.

## Discussion

Occurrence of paroxysmal non-epileptic episodes of frightened facial and body expression, phenotypically similar to PSH following an acute traumatic or anoxic brain injury, is a clinical manifestation that gives rise to great dismay in parents of young people with JNCL disease. The attacks typically occur in situations that—from a behavioral developmental point of view—are normally occurring fear provoking activities in persons having a mental capacity of a toddler hearing loud or unfamiliar noises, rises against gravity, meets strange persons, are left alone by parents, or bothered by visceral discomfort. As emotional and cognitive processes cannot be dissociated, and the cognitive apprehension is critically involved in both emotional experiences and coping strategies (20) we must perceive it unfeasible for adolescent and adult JNCL patients having severe dementia to encounter a normal anxiety response. By referring to psychological, patho-etiological, and neurophysiological studies regarding anxiety- and fear inducing mechanisms it therefore seems well justified that the JNCL patients most probably experience the basic emotion, fear, as a natural response when exposed to the provoking factors. We still need to further clarify the order of the process, and also all the pathophysiological mechanisms behind, but considering the underlying process as the result of a significant intellectual reduction and an autonomic dysfunction is a significant and valid breakthrough how to understand the genesis of these episodes.

The author recognizes limitations to the proposed theory. A very clear distinction between the autonomic symptoms triggered by the actual fear exposure and an eventually autonomic dysfunctions due to the neurodegenerative process itself is difficult to clarify, and the demonstrated concomitant imbalance of the autonomic nervous system may be an epiphenomenon not causative related to the hyperactivity event. However, there are plenty of examples of a relationship between dysfunction of the autonomic nervous system and pathological clinical phenomena in other neurodegenerative diseases. For instance, at the early-mid stages of Huntington's disease an increased sympathetic activity, as detected by HRV measures, is associated with the odds of being a recurrent faller, independent of orthostatic phenomena (45), and in Parkinson the presence of autonomic failure gives rise to higher levels of anxiety (10). Additionally, an increased sympathetic nervous system activity and a decreased parasympathetic nervous system activity have been demonstrated in individuals prone to respond with excessive worry, tension, and physiological arousal when exposed to evaluative stress (46). Moreover, exactly at the pinnacle of the inverted-U-shaped curve describing how amusement and fear coexist in frightening leisure activity as enjoyable until fear takes over, investigations of the heart rate variability show a shift from a parasympathetic dominance to a sympathetic dominance (19). Thus, a real and authentic connection between a fearful emotion and the autonomic nervous system appears realistic and relevant.

The next step in the assessment of how dysfunction of the autonomic nervous system may contribute to the symptomatology of the fearful behavior of young people with JNCL could well be bed-side HRV measurements before, during and after the episodes, eventually combined with HRV measurements during preventive and therapeutic measures. Preferably, these measurements should be accompanied with clinical assessment of presence and severity of excess adrenergic activity and a diagnostic likelihood tool like the PSH Assessment Measure (38) and eventually also by other exploratory approaches to quantify autonomic changes (47).

## Data availability statement

The original contributions presented in the study are included in the article/supplementary material, further inquiries can be directed to the corresponding author.

## Author contributions

The author confirms being the sole contributor of this work and has approved it for publication.
